# Therapy of bilateral vocal fold paralysis: Real world data of an international multi-center registry

**DOI:** 10.1371/journal.pone.0216096

**Published:** 2019-04-29

**Authors:** Tadeus Nawka, Markus Gugatschka, Jan-Constantin Kölmel, Andreas Harald Müller, Berit Schneider-Stickler, Svetlana Yaremchuk, Maria Grosheva, Rudolf Hagen, Joachim T. Maurer, Claus Pototschnig, Thomas Lehmann, Gerd Fabian Volk, Orlando Guntinas-Lichius

**Affiliations:** 1 Department of Audiology and Phoniatrics, Charité University Medicine Berlin, Berlin, Germany; 2 Division of Phoniatrics, Medical University of Graz, Graz, Austria; 3 Department of Otorhinolaryngology, Klinikum Stuttgart, Katharinenhospital, Stuttgart, Germany; 4 Department of Otorhinolaryngology, SRH Wald-Klinikum Gera, Gera, Germany; 5 Division of Phoniatrics-Logopedics, Department of Otolaryngology, Medical University of Vienna, Vienna, Austria; 6 Institute of Otolaryngology of the National Academy of Medical Science of Ukraine, Kiev, Ukraine; 7 Department of Otorhinolaryngology, Head and Neck Surgery, University of Cologne, Cologne, Germany; 8 Department of Otorhinolaryngology, University of Wuerzburg, Wuerzburg, Germany; 9 Department of Otorhinolaryngology, Head and Neck Surgery, University-Hospital Mannheim, Mannheim, Germany; 10 Department of Otorhinolaryngology, University of Innsbruck, Innsbruck, Austria; 11 Institute for Medical Statistics, Computer Science and Data Science, Jena University Hospital, Jena, Germany; 12 Department of Otorhinolaryngology, Jena University Hospital, Jena, Germany; University of Chicago, UNITED STATES

## Abstract

**Purpose:**

To collect data on diagnosis, treatment, patient’s management, and quality of life in patient with bilateral vocal fold paralysis (BVFP).

**Methods:**

A retrospective, observational, multicenter registry study was performed. Medical records of 326 adults with permanent BVFP (median age: 61 years; 70% female, 60% after thyroid surgery) generated between 2010 and 2017.

**Results:**

Median time between BVFP onset and inclusion was 1.2 years. Median post-treatment follow-up was 2 months (range: 0–42). Surgery was treatment of choice in 61.7% of the cases, with a 2-year revision rate of 32.4%. Prior to inclusion, 40.2% of the patients underwent at least one surgery. For tracheotomized patients, decannulation rate was 33.8%. Non-surgical treatments included voice therapy and botulinum toxin injection. Corticosteroid application was the most frequent treatment for post-treatment complications (18%; 1-month after surgery). Older age was an independent predictor for dyspnea (Hazard ratio [HR] = 1.041; CI = 1.005 to 1.079; p = 0.026) and the need for oxygen treatment (HR = 1.098; CI = 1.009 to 1.196; p = 0.031). Current alcohol consumption (HR = 2.565; CI = 1.232 to 5.342; p = 0.012) and a cancer-related etiology (HR = 4.767; CI = 1.615 to 14.067; p = 0.005) were independent factors of higher revision risk.

**Conclusions:**

Surgery for BVFP is currently not standardized but highly variable. Postoperative and BVFP-related complications and revision surgery are frequent. Complications are linked to patients’ alcohol drinking habits and BVFP etiology. These results shall be confirmed by the upcoming evaluation of the prospective data of this registry.

## Introduction

Bilateral vocal fold paralysis (BVFP) is a rare disease with an approximate incidence of 0.95 cases per 100,000 persons [1]. Since this disease occurs so seldom, there is a lack of standardized protocols and outcome measures for its diagnosis, treatment and follow-up [2–4] and consequent high variability among the handling approaches across the various hospitals. In particular, there is a lack of large, systematic studies focused on transoral surgery, which is considered the state-of-the-art therapy for non-life-threatening BVFP cases [2]. Transoral surgery significantly improves the respiratory patency by relieving the symptoms associated with the moderate to severe dyspnea typical of BVFP and increases the decannulation rate in previously tracheotomized patients [5]. However, it also associates with high revision frequency and complications, often requiring patient’s re-hospitalization.

While the risk factors associated with BVFP surgery are well-known to the experts in the field, there is a need of published works in this area, focused on large cohorts, such as patients’ registries. This type of data collection represents the golden standard to collect real-life information on rare diseases and their management. Being this type of registry merely observatory, the results of different standard protocols for diagnosis, treatment and follow-up in use at the various recruiting sites are collected and available for systematic comparison. Thus, we established an international and multi-language BVFP registry aiming to collect this type of data, retrospectively and prospectively, across various sites in Europe.

This first publication focuses on the evaluation of the results and complications of BVFP surgical treatments, based on retrospective data collected across 11 sites in Europe. A later publication based on the same data will focus on functional aspects of BVFP.

## Material and methods

The registry aims to collect real-life data concerning BVFP epidemiology, diagnosis, treatment, patient’s management, and impact of the disease on the quality of life before and after treatment. It is an observatory, non-interventional, systematic, longitudinal, prospective and retrospective, multicentric and multi-language, case series, open-label study, approved by the Ethics Committee of Jena (No. 4530-08/15). The Ethics Committee waived the requirement for informed consent for the patients with exclusive retrospective data analysis using the patients’ charts. The registry is registered in the German Clinical Trials Register (Deutsches Register Klinischer Studien [DRKS], no. DRKS00015028).

Retrospective data were collected between January 2010 and December 2017 in 7 sites in Germany; 3 sites in Austria and 1 site in Ukraine. Detailed information about them is provided in **S1 Table.** The registry procedures and documentation comply with the principles of the Clinical Practice Guidelines, in particular of the ISO 14155:2011, and the Declaration of Helsinki.

Adult subjects of both sexes diagnosed with BVFP were included after signing the informed consent in case of prospective data collection. All the information available on the medical records of each included patient was retrieved, documenting BVFP diagnosis, treatment and 2-year post-treatment follow-up. Particular care was taken to include only patients with neurogenic etiology of BVFP and to exclude patients with non-neurogenic etiology (for instance, posterior glottic stenosis, ankylosis of the cricoarytenoid joint). In short, pseudonymized data on patient’s demographics, medical and medication history (including comorbidity history), BVFP diagnosis, BVFP treatment, and potential post-treatment complications, assessment of the BVFP-related symptoms before and after treatment were recorded. Concerning the surgical procedures, we distinguished between planned surgeries for BVFP (scheduled surgeries) and unplanned surgeries (non-scheduled surgeries) which became necessary due to complications of planned surgeries or due to emergency situations related to BVFP-related symptoms. Such information was documented from the last available medical record before treatment and from the medical records compiled 1 month, 2–4, 5–7, 8–10, 11–13, 14–19, and 20–25 months after treatment.

### Statistical analysis

Only patients showing no sign of spontaneous recovery from BVFP were included for analysis. Patient demographics and outcome variables were analyzed with IBM SPSS statistics software (IBM Corp. Released 2017. IBM SPSS Statistics for Windows, Version 25.0. Armonk, New York) for medical statistics. Data are presented as frequencies or mean ± standard deviation (SD) if not otherwise indicated. Kaplan-Meier curves and log rank tests were used to analyze the probability for revision surgery and for predictors for revision surgery. Multivariable analysis was performed using the Cox proportional hazards model to estimate the hazard ratio (HR) with a confidence interval (CI) of 95% for the revision surgery rate. Only parameters with significant influence on the revision surgery rate in univariable analysis were included in the multivariable analysis. To analyze if age or gender had influence on the occurrence of complications, Cox regression models with the time-dependent covariate surgery were used to detect independent predictors for the complications associated with the BVFP onset vs. with BVFP-related surgical treatment. The significance level was set at p < 0.05.

## Results

### Patients’ characteristics

Three-hundred and twenty-six (326) patients with permanent BVFP were included. Their characteristics at baseline are summarized in **Table 1**. 69.9% of the patients were female and the most common etiology was thyroid surgery (60.4%). The median age at baseline was 61 years. Whereas only 11% of the patients were current smokers, still 27.3% of the patients were drinking alcohol. Almost ¾ of the patients (72.4%) reported an endocrine comorbidity and about half of the patients (54.6%) suffered from a cardiovascular disease. With an average body mass index (BMI) of 25.7, the analyzed BVFP population was graded as overweight. 34/326 patients (10.4%) were tracheotomized at the time of enrolment.

**Table 1:** Patients‘ characteristics at enrollment (N = 326).

Parameter	Absolute number (N)	Relative number (%)
Gender		
Female	228	69.9
Male	98	30.1
Etiology		
Iatrogenic, thyroid surgery	197	60.4
Iatrogenic, other	44	13.5
Idiopathic	42	12.9
Cancer	20	6.1
Traumatic	5	1.5
CNS disorder	6	1.8
Other	5	1.5
Unknown	7	2.1
Comorbidity		
Endocrine	236	72.4
Cardiovascular	178	54.6
Gastrointestinal	126	38.7
Metabolic	111	34.0
Musculoskeletal	91	27.9
Nervous system	67	20.6
Respiratory	65	19.9
Psychological/psychiatric	61	18.7
Urinary/hepatobiliary	58	17.8
Smoking habits		
Never	134	41.1
Formerly	55	16.9
Currently	36	11.0
Unknown	101	31
Alcohol consumption		
Yes	89	27.3
No	237	72.7
Tracheostomy		
Never	267	81.9
Closed	25	7.7
Open	34	10.4
	**Mean ± SD**	**Median (Range)**
Age at onset of BLVP (years)	52.6±18.2	54 (0–91)
Age at registration (years)	59.8±15.2	61 (21–91)
Duration of bilateral paralysis to baseline, years	6.9±12.1	1.2 (0–73)
Body mass index (BMI; kg/m^2^)	26.2±5.9	25.7 (15.2–48.3)

### Treatment

The median time between onset of BVFP and enrolment in the registry was 1.2 years. 131/326 patients (40.2%) received between 1 and 7 BVFP-treatments before being enrolled in the registry. Details on the treatment prior to registration and at baseline are given in **S2 Table**. In 70.7% of the cases the treatment was either surgical glottal enlargement or tracheostomy.

The rate and characteristics of at the baseline and at later check-points are summarized in **Table 2** and **Table 3**. Due to the observatory nature of the registry, the number of patients who underwent a post-treatment follow-up was significantly lower compared to the baseline. Thus, it is impossible to determine whether the need of additional therapies to or revisions surgeries of the treatment(s) the patient underwent at baseline absolutely changed during the evaluated 2-year follow-up period. Although further surgical treatment decreased after initial treatment of BVFP, a small but constant increase of further surgery is seen from 9% (21/233 patients) at 1 month after baseline to 18% (8/45 patients) at 20–25 months after registration.

**Table 2:** Treatment history from baseline to 5–7 months of follow up.

	Baseline		Month 1		Months 2–4		Months 5–7	
Treatment	Absolute number (N)	Relative number (%)	Absolute number (N)	Relative number (%)	Absolute number (N)	Relative number (%)	Absolute number (N)	Relative number (%)
Datasets available	326	100	233	100	180	100	143	100
Main BVFP treatment								
Surgical treatment	201	61.7	21	9.0	21	11.7	17	11.9
Non-surgical treatment only	41	12.6	35	15.0	18	10.0	11	7.7
No treatment	84	25.8	177	76.0	141	78.3	115	80.4
Surgery alone	191	58.6	18	7.7	20	11.1	16	11.2
Surgery + voice therapy	8	2.5	3	1.3	1	0.6	1	0.7
Surgery + other	2	0.6	0	0	0	0	0	0
Voice therapy	39	12	35	15.0	17	9.4	11	7.7
Botulinum toxin	2	0.6	0	0	1	0.6	0	0
Details of surgical treatment								
Glottal enlargement	151	46.3	11	4.7	6	3.3	7	4.9
Glottal enlargement + tracheostomy	4	1.2	1	0.4	5	2.8	3	2.1
Tracheostomy	44	13.5	8	3.4	9	5.0	6	4.2
Glottal medialization	2	0.6	1	0.4	1	0.6	1	0.7

**Table 3:** Treatment history from 8–12 to 20–25 months of follow up.

	Months 8–10		Month 11–13		Month 14–19		Months 20–25	
Treatment	Absolute number (N)	Relative number (%)	Absolute number (N)	Relative number (%)	Absolute number (N)	Relative number (%)	Absolute number (N)	Relative number (%)
Datasets available	119	100	95	100	75	100	45	100
Main BVFP treatment								
Surgical treatment	13	10.9	14	14.7	11	14.7	8	17.8
Non-surgical treatment only	6	5	5	5.3	4	5.3	5	11.1
No treatment	100	84	76	80	60	80	32	71.1
Surgery alone	10	8.4	13	13.7	9	12	8	17.8
Surgery + voice therapy	3	2.5	1	1.1	2	2.7	0	0
Surgery + other	0	0	0	0	0	0	0	0
Voice therapy	5	4.2	4	4.2	4	5.3	4	8.9
Botulinum toxin	1	0.8	1	1.1	0	0	1	2.2
Details of surgical treatment								
Glottal enlargement	8	6.7	5	5.3	7	9.3	5	11.1
Glottal enlargement + tracheostomy	1	0.8	1	1.1	1	1.3	2	4.4
Tracheostomy	4	3.4	8	8.4	3	4	0	0
Glottal medialization	0	0	0	0	0	0	1	2.2

201/326 (61.7%) of the patients received a surgical treatment at baseline. Other non-surgical treatments applied at baseline included voice therapy and botulinum toxin injection.

**Fig 1:**
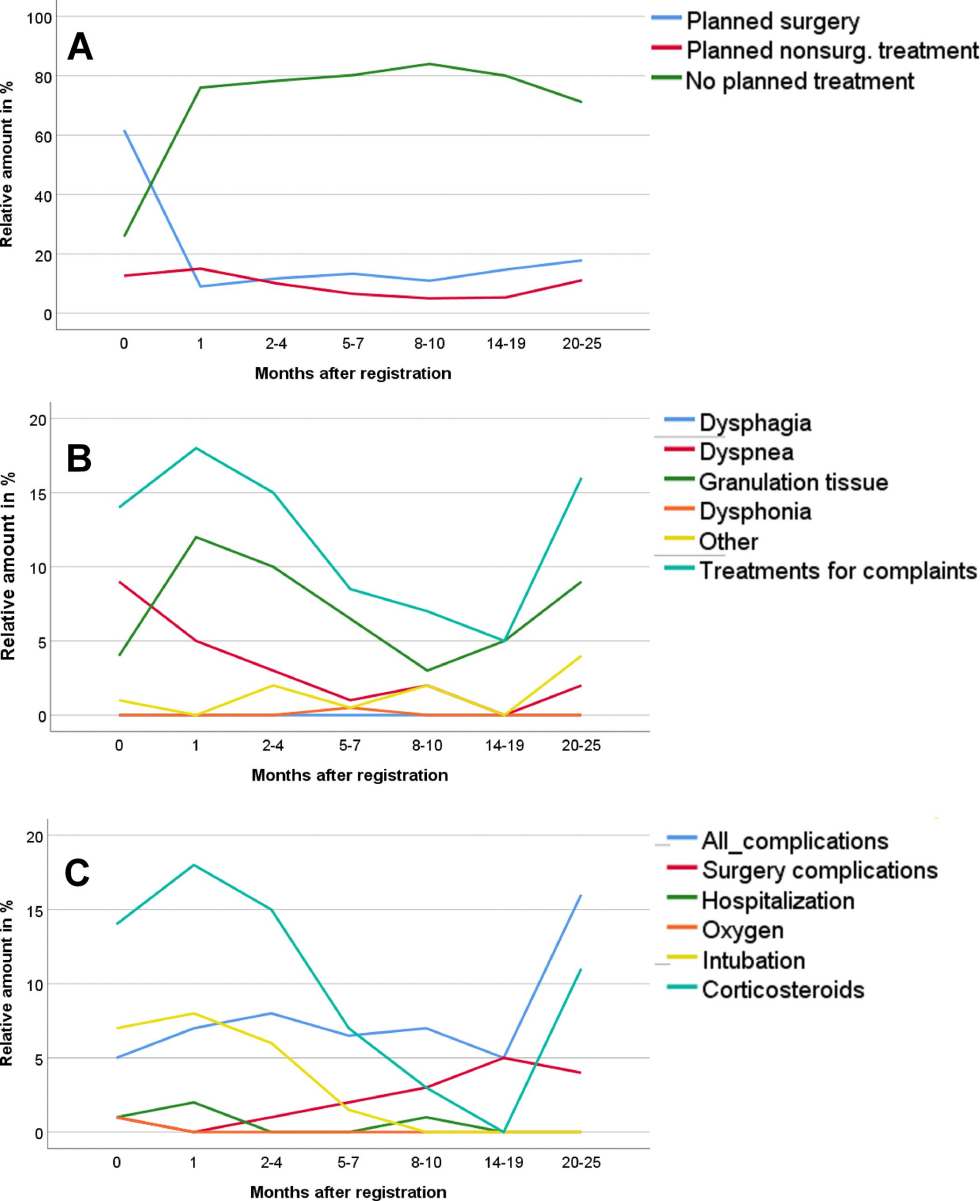
Relative amount of patients with surgery, non-surgical treatment and complications from baseline to 25 months later. **A:** Percentage of patients undergoing scheduled surgical vs. non-surgical BVFP treatment and of patients requiring no BVFP-related treatment from the baseline to the last follow-up visit (max. 25 months after baseline). **B:** Evaluation of the changes in the BVFP symptomology and need of symptom medical treatment from the baseline to the last follow-up visit. **C:** Changes in the detection of BVFP- vs. BVFP treatment-related complications from the baseline to the last follow-up visit. The percentage of patients was always calculated on the total number of patients for which medical acts were available at the respective check-point.

After baseline and within the last follow-up visit, 328 scheduled surgeries and 122 non-scheduled surgeries were performed for a total of 511 surgeries. According to these data, each included BVFP patient was expected to undergo between none and 11 scheduled surgical treatments and between none and 10 non-scheduled surgeries. **Fig 1A** depicts the percentage of patients undergoing scheduled surgical vs. non-surgical BVFP treatment and of patients requiring no BVFP-related treatment from the baseline to the last follow-up visit (max. 25 months after baseline).

The average inpatient treatment duration for any kind of BVFP treatment or for BVFP-related complications was 8.4± 9.6 days. The most frequent surgical approach was the one-sided glottal enlargement (46.3%). Tracheostomy, glottal medialization, or combinations thereof were less frequently used.

Glottal enlargement was performed according to different protocols, mostly depending on the specific experience of the hospital where the procedure was performed (cf. **S2 Table**). In 30 cases, the examined medical records did not provide details about the performed glottal enlargement, thus the latter could not be categorized. In most of the other cases, the performed glottal enlargement was classified as transient laterofixation, posterior cordotomy, partial arytenoidectomy, or as combination of these procedures. Glottal enlargement was performed in 41.6% of the cases on the right and in 37.9% of the cases on the left vocal fold. In one case, both vocal folds underwent surgery.

**Fig 2:**
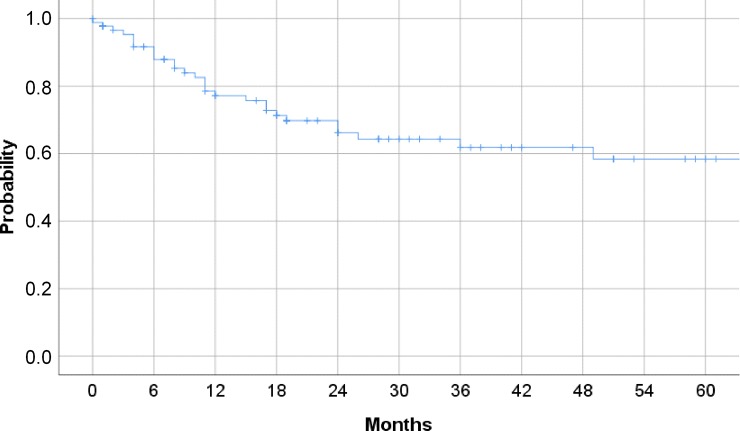
Probability of decannulation. Decannulation rate (Y-axis) of 97 patients, who underwent tracheostomy before or at baseline. Considering the pre-baseline period, the patients were followed up for a maximum of 60 months (X-axis). The Kaplan-Meier curve shows that this rate decreased with time and reached a plateau about 24 months after tracheostomy.

After baseline and within the last follow-up visit, 97 tracheostomies were recorded. The median follow-up time of the tracheotomized patients was 19 months. At this point, only 34/97 (35.0%) patients were decannulated, while 63/97 (64.9%) still required an open tracheostomy. The decannulation rate of at baseline tracheotomized patients was 33.8% and 38.2% at 24 and 48 months, respectively. Considering all the recruited patients who were tracheotomized either before or at baseline, the mean time required for decannulation was 11.7 years (95% confidence interval [CI] = 10.7.77 to 14.5 years). While the probability of decannulation constantly decreased over time, the plateau was reached at about 24 months after its opening (**Fig 2**). We failed to find any decannulation predictors among the examined patient’s or BVFP-treatment characteristics (data not shown).

### BVFP-related and BVFP-treatment-related complications

The patients enrolled in this registry were followed up for a median period of 2.33 [0–42.28] months after baseline. BVFP onset led to moderate to severe dyspnea (**Fig 1B**). This was seen in 9% of the patients after initial treatment before or at baseline and the frequency declined during the further time course (**S3 Table** and **Fig 1B**).

The most common drawback of surgical glottal enlargement was found to be the formation of tissue granulations in correspondence of the surgery site. This issue was observed prevalently 1 month after surgery (12%) and constantly declined over time until 20 months post-treatment follow-up. Between 20 and 25 months after surgery the rate of tissue granulation starts again to increase.

**Table 4** and **Fig 1C** depict the rate of complications related to surgical and non-surgical BVFP treatments. Corticosteroid application was the therapy of choice in 18% of the evaluated patients for surgery-related complications occurring within 1 month after treatment. Need for corticosteroid therapy decreased between 1 and 20 months after surgery and increased again thereafter. Intubation was required to treat BVFP symptoms after primary BVFP treatment in patients not undergoing surgery in 7% of the cases at baseline and 8% of the cases 1 month after registration. With time, the need of this treatment steeply decreased.

**Table 4:** Treatment of BVFP or BVFP-surgery related complications from baseline to 20–25 months of follow up.

	**Baseline**		**Month 1**		**Months 2–4**		**Months 5–7**	
**Treatment**	**Absolute number** **(N)**	**Relative number (%)**	**Absolute number** **(N)**	**Relative number (%)**	**Absolute number** **(N)**	**Relative number (%)**	**Absolute number** **(N)**	**Relative number (%)**
Datasets available	326	100	233	100	180	100	143	100
Patients without complication-related treatments	279	85.6	191	82.0	151	83.9	129	90.2
All complication-related treatments	47	14.4	42	18.0	29	16.1	14	9.8
Surgery because of complications	3	0.9	1	0.4	1	0.6	1	0.7
Hospitalization for surveillance only	2	0.6	4	1.7	0	0	0	0
O_2_ administration	4	1.2	1	0.4	0	0	0	0
Intubation because of severe dyspnea	23	7.1	19	8.2	12	6.7	4	2.8
Corticosteroid treatment	47	14.4	42	18.0	29	16.1	14	9.8
	**Months 8–10**		**Month 11–13**		**Month 14–19**		**Months 20–25**	
	**Absolute number** **(N)**	**Relative number (%)**	**Absolute number** **(N)**	**Relative number (%)**	**Absolute number** **(N)**	**Relative number (%)**	**Absolute number** **(N)**	**Relative number (%)**
Datasets available	119	100	95	100	75	100	45	100
Patients without complication-related treatments	111	93.3	88	92.6	71	94.7	38	84.4
All complication-related treatments	8	6.7	7	7.4	4	5.3	7	15.6
Surgery because of complications	3	2.5	3	3.2	4	5.3	2	4.4
Hospitalization surveillance only	1	0.8	0	0	0	0	0	0
O_2_ administration	0	0	0	0	0	0	0	0
Intubation because of severe dyspnea	0	0	0	0	0	0	0	0
Corticosteroid treatment	4	3.4	4	4.2	0	0	5	11.1

As summarized in **S4 Table**, the Cox regression analyses with time-dependent covariates showed that gender was no predictor for the occurrence of complications. However age was an independent, though weak predictor for dyspnea (Hazard ratio [HR] = 1.041; CI = 1.005 to 1.079; p = 0.026) and the consequent need for oxygen treatment (HR = 1.098; CI = 1.009 to 1.196; p = 0.031).

**Fig 3:**
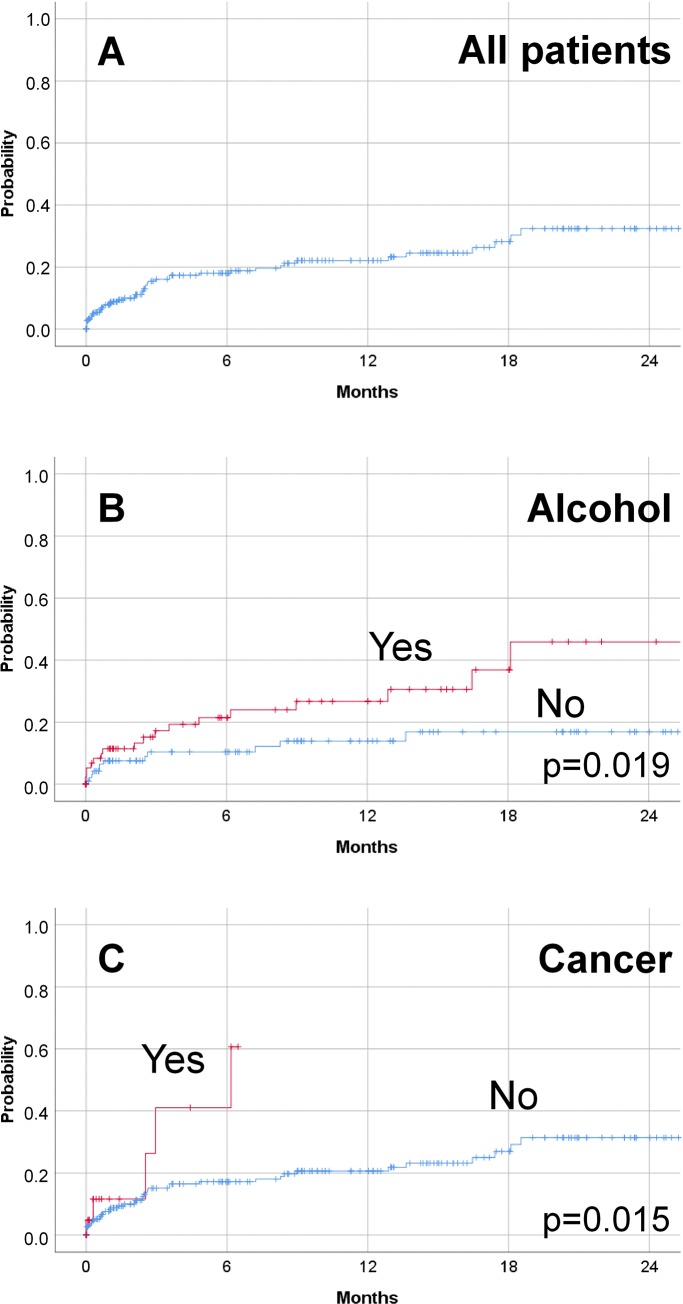
Revision surgery rate for patients who underwent a surgery at baseline. The period considered for analysis is comprised between baseline and the last follow-up visit (max. 25 months after baseline). The revision need probability was calculated by means of the Kaplan-Meier curve. **A:** When all 326 patients were considered for analysis, the probability that a revision is needed increased with time and reached a plateau after 18 months from the first surgery. **B:** Patients regularly drinking alcohol showed a significantly (p = 0.019) increased need of revision surgeries compared with patients who do not drink it. **C:** Patients for whom the BVFP onset is due to a cancer showed a significantly (p = 0.015) increased need of revision surgeries compared with patients who do not drink it.

Considering all the patients included retrospectively in the registry, forty-six (46) revision surgeries were required in the 2-year follow-up period after the first surgery, which implies a revision rate of 32.4% (**Fig 3A**). In this population, alcohol drinking habits and cancer etiology of the BVFP were associated with an increased risk for revision surgeries (p = 0.019; p = 0.015, respectively; **Fig 3B and 3C**). As summarized in **S5 Table** we failed to observe significant associations between the need of revision surgeries and other patients’ characteristics, such as BMI or smoking habits.

However, the multivariable Cox regression analysis confirmed that current alcohol consumption (HR = 2.565; CI = 1.232 to 5.342; p = 0.012) and a cancer-related etiology (HR = 4.767; CI = 1.615 to 14.067; p = 0.005) were independent factors associated with a higher risk of revision surgery.

While we could not see any specific benefits associated with a particular protocol of surgical glottal enlargement in terms of decreased need of revisions, we observed that when the first surgery is performed by means of a single technique (e.g. laterofixation), the need of revisions was significantly (p = 0.023) higher compared with glottal enlargement protocols based on a combination of techniques (e.g. laterofixation + partial arytenoidectomy).

## Discussion

Patient registries are powerful tools for the real-life monitoring of rare diseases and their management in different hospitals. In the case of BVFP, this registry provides systematic information on large cohorts regarding the characteristics of the BVFP patients, their medical history, and most frequent comorbidities, as well as on the most common therapy protocols for this disease and its outcomes. Thus, our results can be used to describe in more details the BVFP care patterns, including appropriateness vs. disparities in its delivery; to assess treatment effectiveness; to monitor safety and harm; and to measure quality of care [6]. This is particularly important seeing the lack of systematic evidence on the epidemiology and health care standards for this rare disease. Since the surgery rate for BVFP is 0.52/100,000 [1], it is not surprising that large cohort prospective or even retrospective trials are missing for this disease (for further details, refer to **S6 Table**).

To the best of our knowledge, the current sample size of this registry (326 patients) constitutes the largest population for which results have been published on permanent BVFP. The international characteristics of this registry allows a systematic comparison of state-of-the-art diagnostic, therapeutic and follow-up approaches as they have been implemented in the last 8 years by various hospitals, while most of the available publications refer to data collected decades ago and thus possibly no longer complying with the current state-of-the-art requirements.

97/326 patients (29.7%) of the patients, were tracheotomized before or during the observation period. In most cases this procedure was chosen because of the life-threating situation of the patients, caused by BVFP-related dyspnea. Decannulation following later transoral surgery was possible in 33.8% of the tracheotomized patients. In the literature, the reported decannulation rate ranges from 11% to 100%, i.e. decannulation rates show a high variability among the published results. This is most likely due to the methodological limitations of the most BVFP studies, in terms of design, sample size, control of biases, recruitment type, and selection criteria [7–11].

The largest retrospective study published so far was a monocentric and included 132 retrospectively included BVFP patients, who underwent posterior cordotomy [10]. In this study, 31% of the patients were tracheotomized before glottal enlargement. The decannulation rate for this group was 63% at the end of the study. The most likely explanation for the discrepancy between the results of this study and those of our registry is that in the former case, the decannulation rate was based on a single recruiting site, while the present study represents the average results of 11 sites across Europe. In addition, while in the former study all the patients underwent posterior cordotomy, the patients enrolled in our registry underwent different types of glottal enlargement.

We showed that BVFP-related dyspnea was not significantly relieved by glottal enlargement in 5% of the patients 1 month and in 3% of the patients 3 months after surgery. The results of our registry showed that age is a significant predictor for post-treatment dyspnea, while it was not found to be reliable predictor for decannulation, which is in contradiction with the results of a previous study [10]. However, the results of our registry confirmed that older patients (≥ 65 years old) required more medical care. In this sub-group, tissue granulation was a frequent side effect of glottal enlargement and affected 12% of the patients 1 month, 10% of the patients between 2 and 4 months, and 8% of the patients between 5 and 7 months after surgery.

We observed that the need for post-surgery pharmacological treatments, such as corticosteroid administration and intubation were quite frequent (overall, 145 corticosteroid treatments and 58 intubations after registration). While in most published works these drawbacks are not accounted for, Brake and Anderson reported that 4% of their patients needed revision surgery because of granulation tissue [7]. Also, the prospective trial of Nawka et al. reported an adverse event rate of 47.2% in the first 6 months after BVFP surgery [2]. Dyspnea was the most frequent complication and was observed in in 45% of their patients. This higher frequency of dyspnea compared to previous works is likely due to the prospective nature of the study, which ensures a more accurate adverse event report than retrospective data collections or case reports.

Severe symptoms at the onset of BVFP or treatment related complication often require surgery or re-surgery. In Nawka et al. [2] patients underwent transoral surgery, mainly posterior cordotomy in combination with partial arytenoidectomy. The revision rate in the 6-month follow-up after the first surgery monitored in that study was 25%, while the 2-year probability of revision surgery calculated based on the results of our registry is 32.4%. Alcohol abuse and cancer related reasons of the BVFP were significant risk factors for revision need. We can only speculate on the reasons. It is well known that risky alcohol drinking is associated with increased postoperative complications [12]. In patients with cancer-related BVFP, it might be that the surgeons are more likely make compromises and do not follow standard surgery, or that revision was needed because the cancer itself has progressed, or cancer-related treatment had changed the situation.

Interestingly, we showed that glottal enlargement based on a combination of different techniques had a lower revision risk than surgery based on a single technique. We assume that this finding could be linked to the fact that procedures based on physiological abduction of the arytenoid cartilage seem to be the most effective [13]. In summary, while surgical glottal enlargement is likely to be more cost-effective than tracheostomy [14], it still show a relevant risk of revision in the 2 years following the first treatment, as also reported by Jackowska et al. [10]. Thus, it is important to consider new, more physiological approaches with a potential low rate of revisions. Selective laryngeal reinnervation surgery and laryngeal pacing show such potentialities although, until now, only in small studies. Larger clinical trials are expected to provide more insights about these innovative techniques [15,16].

The retrospective data analysis provided in this publication has several limitations that shall be addressed. In the first place, while the provided total number of observations is still higher than that reported in any other previous publications, it significantly decreased over the 2-year follow-up, showing that less than 15% of the BVFP patients were followed up for such a long period. Because of this change in the sample size over time it is difficult to calculate the rate of complications over time. Furthermore, BVFP patients with symptoms needing (revision) surgery might have a higher probability to return to the hospital, i.e. there is a higher probability to get follow-up information on this subpopulation. Thus, it might be that we overestimated the complication rate. Analysis of the prospective data might help here, which should guarantee a more stable number of patients over the monitored time.

Because of the extensive amount of data collected in this registry, it was not possible to summarize all the outcomes in a single publication. Functional analysis concerning voice and respiratory parameters will be presented in a following publication.

The final analysis of both retrospective and prospective data should help us to better understand the problems linked to the standardization of diagnosis, treatment and follow-up of BVFP patients and point out the best outcome measures to improve and make the decision making more consistent across the hospitals and countries [3]. The standardization of the evaluation of BVFP treatments would also help defining the outcome measures according to which new approaches, such as like reinnervation surgery or laryngeal pacing should be compared to.

In conclusion, the evaluation of the retrospective data collected in this registry showed that while, as expected, glottal enlargement constitutes the golden standard treatment for BVFP, its protocol is not standardized across hospitals, although our results suggest that the treatment benefits are strictly linked to the choice of the surgical procedure. We also showed that a significant portion of patients still relies on tracheostomy. Both surgical approaches show a significant rate of revision needs within 2 years after the first surgery. The risk of revision is strictly linked to patients’ alcohol drinking habits and BVFP etiology.

Due to the significant changes in the analyzed sample size over the 2-year monitoring period considered by this registry, the results shall be confirmed by the evaluation of the prospective data, which we expect to gather in the next 2 to 3 years.

## Supporting information

S1 Table List of recruiting registry sites in alphabetical order. (DOCX)

S2 Table Treatment history of the recruited patients at baseline (N = 249 treatments in 131 out of 326 patients). (DOCX)

S3 Table Symptomology of the patients who required further treatment between baseline and last follow-up visit. (DOCX)

S4 Table Cox regression models with the time-dependent covariate surgery analyzing the prediction of age and gender for the probability of the occurrence of BVFP or BVFP-related complaints, complications, and complication-related treatments. (DOCX)

S5 Table Association between patients’ and treatment characteristics on the revision surgery rate. Data revealed by Kaplan-Meier statistics and log-rank tests.
(DOCX)

S6 Table Clinical trials on surgery for BVFP^#^ and main outcome. (DOCX)
