# Supplementary material for: Therapy of bilateral vocal fold paralysis: Real world data of an international multi-center registry
Source: PLoS One. 2019 Apr 29;14(4):e0216096. doi: 10.1371/journal.pone.0216096 (PMC6488092; doi:10.1371/journal.pone.0216096)
Supplement: S1 Table — (DOCX) [file pone.0216096.s001.docx]

**S1 Table**

| **S1 Table.** List of recruiting registry sites in alphabetical order |
| --- |
| Department of Audiology and Phoniatrics, Charité University Medicine Berlin, Berlin, Germany |
| Department of Otorhinolaryngology, Head and Neck Surgery, University of Cologne, Cologne, Germany |
| Department of Otorhinolaryngology, SHR Wald-Klinikum Gera, Gera, Germany |
| Division of Phoniatrics, Medical University of Graz, Graz, Austria |
| Department of Otorhinolaryngology, University of Innsbruck, Innsbruck, Austria |
| Department of Otorhinolaryngology, Jena University Hospital, Germany |
| Institute of Otolaryngology of the Academy of Medical Science of Ukraine, Kiev, Ukraine |
| Department of Otorhinolaryngology, Head and Neck Surgery, University-Hospital Mannheim, Mannheim, Germany |
| Department of Otorhinolaryngology, Klinikum Stuttgart, Katharinenhospital, Stuttgart, Germany |
| Division of Phoniatrics-Logopedics, Department of Otolaryngology, Medical University of Vienna, Vienna, Austria |
| Department of Otorhinolaryngology, Plastic, Aesthetic and Reconstructive Head and Neck Surgery, University of Wuerzburg, Wuerzburg, Germany |
