# Supplementary material for: Therapy of bilateral vocal fold paralysis: Real world data of an international multi-center registry
Source: PLoS One. 2019 Apr 29;14(4):e0216096. doi: 10.1371/journal.pone.0216096 (PMC6488092; doi:10.1371/journal.pone.0216096)
Supplement: S2 Table — (DOCX) [file pone.0216096.s002.docx]

**S2 Table**

| **S2 Table.** Treatment history of the recruited patients at baseline (N=249 treatments in 131 out of 326 patients) | | |
| --- | --- | --- |
| **Parameter** | **Absolute number (N)** | **Relative number (%)** |
| **Patients (N=326)** | | |
| Number of treatments prior to registration  0  1  2  3  4  5  6  7 | 195  80  16  11  17  3  0  4 | 59.8  24.5  4.9  3.4  5.2  0.9  0  1.2 |
| **Treatments performed before baseline (N=249)** | | |
| Type of treatment (surgical vs. non-surgical):  Surgical  Non-surgical | 176  73 | 70.7  29.3 |
| Type of treatment (details):  Voice therapy  Tracheostomy  Glottal enlargement (uncategorized)  Transient laterofixation  Posterior cordotomy  Tracheostomy closure  Posterior cordotomy + partial arytenoidectomy  Permanent laterofixation  Laterofixation + partial arytenoidectomy  Complete vocal fold resection  Botulinum toxin  Tracheostomy revision  Posterior. cordotomy + laterofixation + partial arytenoidectomy  Partial arytenoidectomy  Vocal fold medialization  Posterior cordotomy + laterofixation | 67  40  30  25  20  18  11  8  6  6  6  5  2  2  2  1 | 26.9  16.0  12.0  10.0  8.0  7.2  4.4  3.2  2.4  2.4  2.4  2.0  0.8  0.8  0.8  0.4 |
| Any type of rehabilitation after surgery performed before enrolment:  No  Yes | 267  59 | 81.9  18.1 |
