# Supplementary material for: Therapy of bilateral vocal fold paralysis: Real world data of an international multi-center registry
Source: PLoS One. 2019 Apr 29;14(4):e0216096. doi: 10.1371/journal.pone.0216096 (PMC6488092; doi:10.1371/journal.pone.0216096)
Supplement: S3 Table — (DOCX) [file pone.0216096.s003.docx]

**S3 Table**

| **S3 Table.** Symptomology of the patients who required further treatment between baseline and last follow-up visit | | | | | | | | |
| --- | --- | --- | --- | --- | --- | --- | --- | --- |
|  | **Baseline** | | **Month 1** | | **Months 2-4** | | **Months 5-7** | |
| **Symptoms needing treatment** | **Absolute number**  **(N)** | **Relative number (%)** | **Absolute number**  **(N)** | **Relative number (%)** | **Absolute number**  **(N)** | **Relative number (%)** | **Absolute number**  **(N)** | **Relative number (%)** |
| Datasets available | 326 | 100 | 233 | 100 | 180 | 100 | 143 | 100 |
| Dysphagia | 1 | 0 | 1 | 0 | 0 | 0 | 0 | 0 |
| Dyspnea | 30 | 9 | 12 | 5 | 6 | 3 | 1 | 1 |
| Granulation tissue | 14 | 4 | 28 | 12 | 19 | 10 | 12 | 8 |
| Dysphonia | 0 | 0 | 0 | 0 | 0 | 0 | 0 | 0 |
| Other | 2 | 1 | 1 | 0 | 4 | 2 | 1 | 1 |
| Total number of symptoms needing treatment | 47 | 14 | 42 | 18 | 29 | 15 | 14 | 10 |
|  | | | | | | | | |
|  | **Months 8-10** | | **Month 11-13** | | **Month 14-19** | | **Months 20-25** | |
|  | **Absolute number**  **(N)** | **Relative number (%)** | **Absolute number**  **(N)** | **Relative number (%)** | **Absolute number**  **(N)** | **Relative number (%)** | **Absolute number**  **(N)** | **Relative number (%)** |
| Datasets available | 119 | 100 | 95 | 100 | 75 | 100 | 45 | 100 |
| Dysphagia | 0 | 0 | 0 | 0 | 0 | 0 | 0 | 0 |
| Dyspnea | 2 | 2 | 1 | 1 | 0 | 0 | 1 | 2 |
| Granulation tissue | 4 | 3 | 5 | 5 | 4 | 5 | 4 | 9 |
| Dysphonia | 0 | 0 | 1 | 1 | 0 | 0 | 0 | 0 |
| Other | 2 | 2 | 0 | 0 | 0 | 0 | 2 | 4 |
| Total number of symptoms needing treatment | 8 | 7 | 7 | 7 | 4 | 5 | 7 | 16 |
