# Supplementary material for: Therapy of bilateral vocal fold paralysis: Real world data of an international multi-center registry
Source: PLoS One. 2019 Apr 29;14(4):e0216096. doi: 10.1371/journal.pone.0216096 (PMC6488092; doi:10.1371/journal.pone.0216096)
Supplement: S4 Table — (DOCX) [file pone.0216096.s004.docx]

**S4 Table**

| **S4 Table.** Cox regression models with the time-dependent covariate surgery analyzing the prediction of age and gender for the probability of the occurrence of BVFP or BVFP-related complaints, complications, and complication-related treatments | | | | |
| --- | --- | --- | --- | --- |
| **Parameter** | **HR** | **Lower 95% CI** | **Upper 95% CI** | **p** |
| **Model 1: Granulation tissue formation** | | | | |
| Re-surgery | 2.643 | 0.808 | 8.642 | 0.108 |
| Age | 1.016 | 0.977 | 1.036 | 0.104 |
| Gender, male | 1.436 | 0.753 | 2.738 | 0.272 |
| **Model 2: Dyspnea** | | | | |
| Re-Surgery | 0.976 | 0.215 | 4.423 | 0.975 |
| Age | 1.041 | 1.005 | 1.079 | **0.026** |
| Gender, male | 0.897 | 0.340 | 2.363 | 0.825 |
| **Model 3: Corticosteroid treatment** | | | | |
| Re-surgery | 3.961 | 1.741 | 9.014 | **0.001** |
| Age | 1.005 | 0.985 | 1.026 | 0.632 |
| Gender, male | 1.110 | 0.559 | 2.206 | 0.765 |
| **Model 4: Oxygen treatment** | | | | |
| Re-surgery | 3,369 | 0,339 | 33,514 | 0.300 |
| Age | 1.098 | 1.009 | 1.196 | **0.031** |
| Gender, male | 2.004 | 0.229 | 17.571 | 0.530 |
| **Model 5: Hospitalization required for complication other than revision surgery** | | | | |
| Re-surgery | 25.064 | 1.950 | 322.190 | 0.013 |
| Age | 1.073 | 0.983 | 1.171 | 0.114 |
| Gender, male | 0.196 | 0.030 | 1.264 | 0.087 |

HR = hazard ratio, CI = confidence interval; significant values (p<0.05) in bold.
