# Supplementary material for: Therapy of bilateral vocal fold paralysis: Real world data of an international multi-center registry
Source: PLoS One. 2019 Apr 29;14(4):e0216096. doi: 10.1371/journal.pone.0216096 (PMC6488092; doi:10.1371/journal.pone.0216096)
Supplement: S5 Table — Data revealed by Kaplan-Meier statistics and log-rank tests. (DOCX) [file pone.0216096.s005.docx]

**S5 Table**

| **S5 Table.** Association between patients’ and treatment characteristics on the revision surgery rate. Data revealed by Kaplan-Meier statistics and log-rank tests | | |
| --- | --- | --- |
| **Parameter** | **2-year re-surgery rate** | **p** |
| All | 32.4% |  |
| Gender  Male  Female | 23.6  36.4 | 0.483 |
| Age  < 61 years (median age)  > 61 years | 30.4  34.8 | 0.464 |
| Currently smoking  No  Yes | 31.0  28.5 | 0.524 |
| Currently drinking alcohol  No  Yes | 26.9  45.9 | **0.019** |
| BMI  Not obese (BMI ≤30)  Obese (BMI>30) | 23.4  27.1 | 0.312 |
| Cancer-related BVFP  No  Yes | 31.4  60.7 | **0.015** |
| Iatrogenic origin of the BVFP  No  Yes | 26.0  35.4 | 0.677 |
| Idiopathic origin of the BVFP  No  Yes | 34.4  16.5 | 0.286 |
| Ever tracheotomized  No  Yes | 29.2  52.0 | 0.437 |
| Primary treatment  At day of BVFP onset  Later | 27.4  40.2 | 0.874 |
| Any BVFP-related surgery at or before baseline  No  Yes | 25.6  34.7 | 0.074 |
| Primary treatment was surgery  No  Yes | 19.4  34.4 | 0.204 |
| If the primary treatment was surgery, was it glottal enlargement  No  Yes | 26.4  35.0 | 0.104 |
| If the primary treatment was glottal enlargement, enlargement was performed by  Single technique (e.g. laterofixation)  Combination of techniques (e.g. laterofixation + partial arytenoidectomy) | 45.0  19.7 | **0.023** |
| If the primary surgery was glottal enlargement, the target vocaol fold was  Left side  Right side | 36.3  36.9 | 0.762 |

BMI = body mass index; Significant values (p<0.05) in bold.
