# Supplementary material for: Therapy of bilateral vocal fold paralysis: Real world data of an international multi-center registry
Source: PLoS One. 2019 Apr 29;14(4):e0216096. doi: 10.1371/journal.pone.0216096 (PMC6488092; doi:10.1371/journal.pone.0216096)
Supplement: S6 Table — (DOCX) [file pone.0216096.s006.docx]

**S6 Table**

**Clinical trials from 2004 to 2018 on surgery for BVFP**

Update of Supplemental Table II in Nawka et al. Permanent transoral surgery of bilateral vocal fold paralysis: a prospective multi-center trial. Laryngoscope. 2015 Jun;125(6):1401-8.

| **S6 Table. Clinical trials on surgery for BVFP^#^ and main outcome** | | | | | | |
| --- | --- | --- | --- | --- | --- | --- |
| **Category** | **Study** | **Design** | **N** | **Surgery** | **Follow-up** | **Main outcome^§^** |
| **Prospective trials** | | | | | | |
| Endolaryngeal surgery | Eckel et al 1994[^1^](#_ENREF_1) | Prospective? | 28 | 18 patients with cordectomy, and 10 patients with arytenoidectomy. | 6-35 months | - Spirometry equal - Phonation time equally decreased - Subclinical aspiration only after arytenoidectomy (50%) - 67% success rate |
| Endolaryngeal surgery | Dursun and Gökcan 2006[^2^](#_ENREF_2) | Prospective? | 22 | Posterior cordotomy | 1 year | - stable airway was achieved with acceptable voice quality and no aspiration in 68.2% - revision surgery 27.2% |
| Endolaryngeal surgery | Yilmaz 2012[^3^](#_ENREF_3) | Prospective? | 50 | Total arytenoidectomy | 1-? years | - All VHI-30 results increased - All aerodynamic results worsened - All acoustic results worsened - revision surgery 6% |
| Endolaryngeal surgery | Yilmaz et al. 2013[^4^](#_ENREF_4) | Prospective, randomized | 10+10 = 20 | 10 patients with total arytenoidectomy and 10 patients with partial arytenoidectomy | 1 year | - Total arytenoidectomy faster - VHI-30 not different - Phonatory and swallowing parameters not different - 100% success rate - 0% revision surgery |
| Endolaryngeal surgery | Gorphe et al. 2013[^5^](#_ENREF_5) | Prospective?* | 20 | Medial arytenoidectomy | 3 months | - Jitter and shimmer worse (N=8) - VHI-120 unchanged - Spirometry unchanged (N=6) |
| Endolaryngeal surgery | Mohamed et al. 2013[^6^](#_ENREF_6) | Prospective, randomized | 10+10 = 20 | Laser-assisted posterior cordotomy versus diathermy-assisted posterior cordotomy | 1 year | - Dyspnea severity scale lower in laser group at 1 year - Dysphonia equal at 1 year - Maximal phonation time longer in laser group at 1 year - 10% revision surgery in diathermy group |
| Endolaryngeal surgery | Hyodo et al. 2008[^7^](#_ENREF_7) | Prospective? | 6+5 = 11 | 6 patients with conventional laterofixation versus 5 patients with new needle carrier | 3 months to 6.7 years | - Spirometry improved - Phoniatry parameters slightly decreased - 90.9% success rate - 9.1% revision surgery |
| Endolaryngeal surgery | Nawka et al. 2015[^8^](#_ENREF_8) | Prospective | 36 | posterior cordotomy, partial arytenoidectomy, laterofixation | 6 months | - Quality of life (SF36) improved - Total VHI unchanged |
| Endolaryngeal surgery | Nawka et al. 2015[^9^](#_ENREF_9) | Prospective | 36 | posterior cordotomy, partial arytenoidectomy, laterofixation | 6 months | - Respiration improved - Voice quality lower |
| Transcervical surgery | Müller et al.2016[^10^](#_ENREF_10) | Prospective | 9 | Laryngeal pacing | 6 months | - 89% Implantation successful - Respiration improved - Quality of life (SF36, GBI) improved - 22% prior tracheostomies - 100% decannulation |
| Transcervical surgery | Müller et al.2017[^11^](#_ENREF_11) | Prospective | 9 | Laryngeal pacing | 6 months | - Voice quality unchanged - Sound pressure level improved |
| **Systematic reviews and meta-analyses** | | | | | | |
| Endolaryngeal + Transcervical surgery | Chen et al. 2014[^12^](#_ENREF_12) | Meta-analysis | 39 studies  1798 | Unilateral and bilateral palsies included  Glottic enlargement or reinnervation surgery | -- | - Type of surgery and timing highly variable - Reinnervation surgery might be better for late surgery >12 months after onset |
| **Retrospective trials** | | | | | | |
| Endolaryngeal surgery | Elsherief et al . 2004[^13^](#_ENREF_13) | Retrospective | 13 | Posterior cordotomy | 6 months to 2.5 years | - Spirometry improved - 100% success rate - 0% revision surgery - Acoustic parameters unchanged |
| Endolaryngeal surgery | Bizakis et al . 2004[^14^](#_ENREF_14) | Retrospective | 18 | Posterior with total arytenoidectomy | ? | - 100% success rate - 0% revision surgery |
| Endolaryngeal surgery | Bosley et al . 2005[^15^](#_ENREF_15) | Retrospective | 11+6 = 17 | 11 patients with posterior cordotomy vs 6 patients with medial arytenoidectomy | 4 months to 5 years | - VHI-10 decrease higher after arytenoidectomy - 47% revision surgery |
| Endolaryngeal surgery | Khalifa et al. 2005[^16^](#_ENREF_16) | Retrospective | 22 | Bilateral posterior cordectomy | ? | - Success rate: 92% - 18% revision surgery |
| Endolaryngeal surgery | Olthoff et al. 2005[^17^](#_ENREF_17) | Retrospective | 17 | Bilateral posterior cordectomy | 1 month to 5 years | - 23.5% revision surgery - Respiratory function subjectively improved - Spirometry improved - RBH voice function decreased |
| Endolaryngeal surgery | Al-Fattah et al. 2006[^18^](#_ENREF_18) | Retrospective | 45 | Partial arytenoidectomy and posterior cordotomy | 2 years? | - 6.7% revision surgery - No pre- to postoperative comparison of functional results |
| Endolaryngeal surgery | Misiolek et al. 2007[^19^](#_ENREF_19) | Retrospective | 30 | Total arytenoidectomy and posterior cordectomy | 5 years | - Ventilation decreased |
| Endolaryngeal surgery | Bajaj et al. 2009[^20^](#_ENREF_20) | Retrospective | 9 | Bilateral posterior cordotomy | 3 months to 4 years | - 0% revisions surgery - Voice symptom scale subjectively good - University of Washington Quality of Life questionnaire with high quality of life |
| Endolaryngeal surgery | Özdemir et al. 2013[^21^](#_ENREF_21) | Retrospective | 66 | Posterior cordotomy | 3 months | - Revision surgery 12% - VHI-10 unchanged |
| Endolaryngeal surgery | Ezzat et al. 2010[^22^](#_ENREF_22) | Retrospective | 21 | Laterofixation | 1 year? | - 95.2% success rate - 19% revision surgery |
| Endolaryngeal surgery | Rovo et al. 2010[^23^](#_ENREF_23) | Retrospective | 22 | Laterofixation | 1-4 years | - Spirometry improved - Some patients with recovery of vocal cord movement - Phoniatric parameters unchanged |
| Endolaryngeal + Transcervical surgery | Brake and Anderson 2015[^24^](#_ENREF_24) | Retrospective | 46 | Tracheotomy in 40%  Posterior cordotomy or arytenoidectomy or medialization thyroplasty | ? | - 15% decannulated - 5% tracheotomy dependent - 4% Granuloma formation - 11% persistent symptoms - 33% revision surgery |
| Endolaryngeal surgery | Howell et al. 2017[^25^](#_ENREF_25) | Retrospective | 6 | Laterofixation | ? | - ? |
| Endolaryngeal surgery | Wiegand et al.[^26^](#_ENREF_26) | Retrospective | 27 | Laterofixation, posterior cordotomy | 1 day to 38 years | - Voice function acceptable - 11% later tracheostomy - 11% decannulation rate of prior tracheotomized patients - ?% revision surgery |
| Endolaryngeal surgery | Su et al. 2014[^27^](#_ENREF_27) | Retrospective | 20 | Laterofixation |  | - 18% revision surgery - Voice acceptable - 10% failure - Prior 15% tracheostomy - 66% decannulation rate |
| Endolaryngeal surgery | Asik et al. 2016[^28^](#_ENREF_28) | Retrospective | 11 | Posterior cordotomy | ? | - Aerodynamic results improved - Voice not significantly decreased - Voice Handicap Index improved - 0% revision surgery - 0% tracheotomies |
| Endolaryngeal surgery | Jackowska et al. 2018[^29^](#_ENREF_29) | Retrospective | 132 | Posterior cordectomy |  | - 63% decannulation rate - 1-3 procedures needed - More procedures needed in non-tracheotomized patients - Advanced age and comorbidity negative predictors |
| Endolaryngeal surgery | Yimaz 2018[^30^](#_ENREF_30) | Retrospective | 64 | Partial arytenoidectomy | ? | - Prior 14% tracheostomy - 100% decannulation rate - Voice Handicap Index improved - Aerodynamic results improved - Increased breathiness |
| Endolaryngeal + Transcervical surgery | Songu et al. 2013[^31^](#_ENREF_31) | Retrospective | 26 | Laterofixation through thyroplasty window | Mean 2 years? | - Voice deteriorated (Likert scale) - Dyspnea improved (MRC dyspnea scale |
| Transcervical surgery | Woodson 2010[^32^](#_ENREF_32) | Retrospective | 11 | Arytenoid abduction | ? | - 63.6% success rate |
| Transcervical surgery | Li et al. 2013[^33^](#_ENREF_33) | Retrospective | 44 | Bilateral PCA muscles reinnervation using the left phrenic nerve | 1 year? | - 87% reinnervation success rate - Voice unchanged - Maximal phonation time unchanged - Spirometry improved |
| Transcervical surgery | Li et al. 2013[^34^](#_ENREF_34) | Retrospective | 5+12 = 17 | 5 patients with laryngeal pacing and 12 patients with posterior cordotomy | ? | - Spirometry better in pacing group - Voice according to voice grade on a 4-point scale better in pacing group |

?=study design not clearly described;  ^#^Retrospective studies only from 2004 to 2014; *Exact study design, primary endpoints and/or alignment to GCP criteria unclear; ^$^Success rate related to decannulation only given for studies with tracheotomized patients

**BIBLIOGRAPHY**

1. Eckel HE, Thumfart M, Wassermann K, Vossing M, Thumfart WF. Cordectomy versus arytenoidectomy in the management of bilateral vocal cord paralysis. The Annals of otology, rhinology, and laryngology 1994; 103:852-857.

2. Dursun G, Gokcan MK. Aerodynamic, acoustic and functional results of posterior transverse laser cordotomy for bilateral abductor vocal fold paralysis. The Journal of laryngology and otology 2006; 120:282-288.

3. Yilmaz T. Endoscopic total arytenoidectomy for bilateral abductor vocal fold paralysis: a new flap technique and personal experience with 50 cases. The Laryngoscope 2012; 122:2219-2226.

4. Yilmaz T, Suslu N, Atay G, Ozer S, Gunaydin RO, Bajin MD. Comparison of voice and swallowing parameters after endoscopic total and partial arytenoidectomy for bilateral abductor vocal fold paralysis: a randomized trial. JAMA otolaryngology-- head & neck surgery 2013; 139:712-718.

5. Gorphe P, Hartl D, Primov-Fever A, Hans S, Crevier-Buchman L, Brasnu D. Endoscopic laser medial arytenoidectomy for treatment of bilateral vocal fold paralysis. European archives of oto-rhino-laryngology : official journal of the European Federation of Oto-Rhino-Laryngological Societies 2013; 270:1701-1705.

6. Mohamed NN, Sorour SS, El-Anwar MW, Quriba AS, Mahdy MA. Comparison between laser- and diathermy-assisted posterior cordotomy for bilateral vocal cord abductor paralysis. JAMA otolaryngology-- head & neck surgery 2013; 139:923-930.

7. Hyodo M, Nishikubo K, Motoyoshi K. Laterofixation of the vocal fold using an endo-extralaryngeal needle carrier for bilateral vocal fold paralysis. Auris, nasus, larynx 2009; 36:181-186.

8. Nawka T, Sittel C, Gugatschka Met al. Permanent transoral surgery of bilateral vocal fold paralysis: a prospective multi-center trial. The Laryngoscope 2015; 125:1401-1408.

9. Nawka T, Sittel C, Arens Cet al. Voice and respiratory outcomes after permanent transoral surgery of bilateral vocal fold paralysis. The Laryngoscope 2015; 125:2749-2755.

10. Mueller AH, Hagen R, Foerster G, Grossmann W, Baumbusch K, Pototschnig C. Laryngeal pacing via an implantable stimulator for the rehabilitation of subjects suffering from bilateral vocal fold paralysis: A prospective first-in-human study. The Laryngoscope 2016; 126:1810-1816.

11. Mueller AH, Hagen R, Pototschnig Cet al. Laryngeal pacing for bilateral vocal fold paralysis: Voice and respiratory aspects. The Laryngoscope 2017; 127:1838-1844.

12. Chen X, Wan P, Yu Yet al. Types and timing of therapy for vocal fold paresis/paralysis after thyroidectomy: a systematic review and meta-analysis. Journal of voice : official journal of the Voice Foundation 2014; 28:799-808.

13. Elsherief S, Elsheikh MN. Endoscopic radiosurgical posterior transverse cordotomy for bilateral median vocal fold immobility. The Journal of laryngology and otology 2004; 118:202-206.

14. Bizakis JG, Papadakis CE, Karatzanis ADet al. The combined endoscopic CO(2) laser posterior cordectomy and total arytenoidectomy for treatment of bilateral vocal cord paralysis. Clinical otolaryngology and allied sciences 2004; 29:51-54.

15. Bosley B, Rosen CA, Simpson CB, McMullin BT, Gartner-Schmidt JL. Medial arytenoidectomy versus transverse cordotomy as a treatment for bilateral vocal fold paralysis. The Annals of otology, rhinology, and laryngology 2005; 114:922-926.

16. Khalifa MC. Simultaneous bilateral posterior cordectomy in bilateral vocal fold paralysis. Otolaryngology--head and neck surgery : official journal of American Academy of Otolaryngology-Head and Neck Surgery 2005; 132:249-250.

17. Olthoff A, Zeiss D, Laskawi R, Kruse E, Steiner W. Laser microsurgical bilateral posterior cordectomy for the treatment of bilateral vocal fold paralysis. The Annals of otology, rhinology, and laryngology 2005; 114:599-604.

18. Al-Fattah HA, Hamza A, Gaafar A, Tantawy A. Partial laser arytenoidectomy in the management of bilateral vocal fold immobility: a modification based on functional anatomical study of the cricoarytenoid joint. Otolaryngology--head and neck surgery : official journal of American Academy of Otolaryngology-Head and Neck Surgery 2006; 134:294-301.

19. Misiolek M, Ziora D, Namyslowski Get al. Long-term results in patients after combined laser total arytenoidectomy with posterior cordectomy for bilateral vocal cord paralysis. European archives of oto-rhino-laryngology : official journal of the European Federation of Oto-Rhino-Laryngological Societies 2007; 264:895-900.

20. Bajaj Y, Sethi N, Shayah Aet al. Vocal fold paralysis: role of bilateral transverse cordotomy. The Journal of laryngology and otology 2009; 123:1348-1351.

21. Ozdemir S, Tuncer U, Tarkan O, Kara K, Surmelioglu O. Carbon dioxide laser endoscopic posterior cordotomy technique for bilateral abductor vocal cord paralysis: a 15-year experience. JAMA otolaryngology-- head & neck surgery 2013; 139:401-404.

22. Ezzat WF, Shehata M, Kamal I, Riad MA. Adjustable laterofixation of the vocal fold in bilateral vocal fold paralysis. The Laryngoscope 2010; 120:731-733.

23. Rovo L, Madani S, Sztano Bet al. A new thread guide instrument for endoscopic arytenoid lateropexy. The Laryngoscope 2010; 120:2002-2007.

24. Brake MK, Anderson J. Bilateral vocal fold immobility: a 13 year review of etiologies, management and the utility of the Empey index. Journal of otolaryngology - head & neck surgery = Le Journal d'oto-rhino-laryngologie et de chirurgie cervico-faciale 2015; 44:27.

25. Howell R, Romeo S, Myer Ct, Bowen M, Khosla S. The lasso technique for endoscopic suture lateralization in bilateral vocal fold immobility. The Laryngoscope 2017; 127:2604-2607.

26. Wiegand S, Teymoortash A, Hanschmann H. Endo-extralaryngeal Laterofixation of the Vocal Folds in Patients with Bilateral Vocal Fold Immobility. In vivo 2017; 31:1159-1162.

27. Su WF, Liu SC, Tang WS, Yang MC, Lin YY, Huang TT. Suture lateralization in patients with bilateral vocal fold paralysis. Journal of voice : official journal of the Voice Foundation 2014; 28:644-651.

28. Asik MB, Karasimav O, Birkent H, Merati AL, Gerek M, Yildiz Y. Impact of unilateral carbon dioxide laser posterior transverse cordotomy on vocal and aerodynamic parameters in bilateral vocal fold paralysis. The Journal of laryngology and otology 2016; 130:373-379.

29. Jackowska J, Sjogren EV, Bartochowska A, Czerniejewska-Wolska H, Piersiala K, Wierzbicka M. Outcomes of CO2 laser-assisted posterior cordectomy in bilateral vocal cord paralysis in 132 cases. Lasers Med Sci 2018; 33:1115-1121.

30. Yilmaz T. Endoscopic Partial Arytenoidectomy for Bilateral Vocal Fold Paralysis: Medially Based Mucosal Flap Technique. Journal of voice : official journal of the Voice Foundation 2018.

31. Songu M, Aslan H, Denizoglu Iet al. Vocal and ventricular fold lateralization using crossing sutures with the thyroplasty window technique for bilateral vocal fold immobility: long-term results. Acta oto-laryngologica 2013; 133:1201-1206.

32. Woodson G. Arytenoid abduction: indications and limitations. The Annals of otology, rhinology, and laryngology 2010; 119:742-748.

33. Li M, Chen S, Zheng Het al. Reinnervation of bilateral posterior cricoarytenoid muscles using the left phrenic nerve in patients with bilateral vocal fold paralysis. PloS one 2013; 8:e77233.

34. Li Y, Pearce EC, Mainthia Ret al. Comparison of ventilation and voice outcomes between unilateral laryngeal pacing and unilateral cordotomy for the treatment of bilateral vocal fold paralysis. ORL; journal for oto-rhino-laryngology and its related specialties 2013; 75:68-73.
